# Reliability and Use of Copenhagen Burnout Inventory in Italian Sample of University Professors

**DOI:** 10.3390/ijerph15081708

**Published:** 2018-08-09

**Authors:** Cristina Sestili, Stefania Scalingi, Sara Cianfanelli, Alice Mannocci, Angela Del Cimmuto, Simone De Sio, Massimiliano Chiarini, Marco Di Muzio, Paolo Villari, Maria De Giusti, Giuseppe La Torre

**Affiliations:** 1 Department of Public Health and Infectious Diseases, Sapienza University of Rome, Piazzale Aldo Moro 5, 00185 Rome, Italy; Stefania.scalingi@libero.it (S.S.); sara.cianfanelli@uniroma1.it (S.C.); alice.mannocci@uniroma1.it (A.M.); angela.delcimmuto@uniroma1.it (A.D.C.); massimiliano.chiarini@uniroma1.it (M.C.); paolo.villari@uniroma1.it (P.V.); maria.degiusti@uniroma1.it (M.D.G.); giuseppe.latorre@uniroma1.it (G.L.T.); 2 Research Unit of Occupational Medicine, Sapienza University of Rome, 00185 Rome, Italy; simone.desio@uniroma1.it; 3 Department of Clinical and Molecular Medicine, Sapienza University of Rome, 00189 Rome, Italy; marco.dimuzio@uniroma1.it

**Keywords:** burnout, academics, stress, prevention, Copenhagen Burnout Inventory

## Abstract

Academics often have to face with burnout syndrome at work. This cross-sectional study evaluates the reliability of the Italian version of the Copenhagen Burnout Inventory (CBI) in a sample of Academics of Sapienza University of Faculty of Medicine and Pharmacy, through an online questionnaire composed of the CBI, SF12 Health Survey, and Positivity Scale. Univariate, bivariate, multivariate analyses, and Cronbach α coefficients of CBI were performed. Ninety-five participants completed the questionnaire (response rate 85%). Cronbach’s α of the three domains were high (0.892, 0.868, and 0.836). Women, younger and part time professors reported higher score in personal (*p* = 0.025; 0.060) and work burnout. In multivariate analysis decreasing age (β = −0.263; *p* = 0.001); being a professor in environmental technicians (β = −0.120; *p* = 0.098); and low mental (β = −0.263; *p* = 0.020), physical (β = −0.319; *p* ≤ 0.001) and positivity scores (β = −0.237; *p* = 0.031) predict significantly higher personal burnout. Low physical (β = −0.346; *p* < 0.001) and mental (β = − 0.249; *p* = 0.013) positivity (β = −0.345; *p* = 0.001) scores; fewer years of work (β = −0.269; *p* ≤ 0.001); and being a medical or nursing professor (β = 0.169; *p* = 0.016) predicts high work burnout. Low MCS predicts a high level of student burnout. Results suggest that the Italian version of the CBI is a reliable instrument. Further research should focus on the prevalence of burnout in academics.

## 1. Introduction

Burnout has different definitions, but the most commonly used is “a state of physical, emotional or mental exhaustion caused by long-term involvement in situations that are emotionally demanding” [1].

The origin of the term ‘burnout’ dates back to in 1970. At the beginning, it was used predominately in popular culture and this root in the society explains the suggestive expression to address such phenomenon [2]. This condition was focused on by sociological sciences afterwards, and subsequently, it started to been investigated in empirical researches based on the scientific theories emerging in concert. Burnout syndrome was initially associated with health and welfare professions (health workers, social services workers, criminal justice employees, teachers, etc.) denominated ‘people work’ and it has been evaluated mainly through a questionnaire called Maslach Burnout Inventory (MBI). The MBI aims at investigating this condition among employees in human service and it operationalizes the definition of the burnout syndrome as a result of three aspects: emotional exhaustion, depersonalization, and lack of personal fulfilment [3,4,5]. This condition was then recognized as being associated with any work context which provides highly stressful and pressing conditions such as positions of high work responsibility and intense involvement independently of the relationship with other people.

Burnout is a notable concern in research due to the negative effects on workers and workplace with consequent social and economic repercussions. The individual outcomes of this phenomenon regard job performance and health. Concerning job performance, burnout results in a lack of job fulfilment, reduction of devotion to job and it is associated to absenteeism, early retirement, and recurrent replacement of work staff. With respect to health outcomes, this syndrome has been linked to mental health disorders such as anxiety, depression, and low self-esteem. Burnout of workers affects also the entire work environment, raising deterioration in the relationships with colleagues, with a consequent worsening of the arrangement of the workflow and the decline of the efficacy and productivity of the work in team [2,6,7,8,9]. Previous studies focused on burnout syndrome among workers in educational institutions, in particular, many authors studied this condition among teachers [10,11,12,13] and its consequences on teachers themselves, in terms of health and efficiency at work, but also on student outcomes. As a consequence of teacher burnout, students more frequently experience negative social and emotional attitude and achieve less goals in their schooling [14,15,16]. From the beginning of the investigation on burnout, school teaching was considered an issue of main interest, due to the demanding kind of work especially for close interaction with students as children and adolescents [17].

Moreover, school teaching is undergoing many changes, concurrently with wide changes in the society, as the traditional family model transforms, along with the development of new technologies, and their application also in the education programmes and the reforms of educational system. Consequently, teachers are exposed to greater emotional challenges that jeopardize their wellbeing and the efficacy and effectiveness of teaching [18,19,20,21]. Academic teaching differs significantly from school teaching, the kind of tasks concerning this profession are different and pertain to faculty and department organization, scientific research, ethical committee issues, planning of the academic year, management of courses with classes and examining sessions, not least, the interaction with adult clients, university students. The main aspects reported in literature are excessive workload, department budget deficits, and disputes with the dean [22,23,24,25]. Another factor reported related to burnout can be the pressure to publish [26]. Scientific publication increases the rank of faculty and of academics and is involved with the university careers of individual academics.

In literature, studies on burnout conducted exclusively on university professors are fewer, with even less research about medical professors. Medical professors have a central position in the medical community, they represent for students, residents, and colleagues an ideal model to be aspire to and, at the same time, they are in charge of management workload in addition to educational and research activities [26].

Some authors investigated burnout in professors in the medicine faculty of specific departments: Gabbe et al. [23] in 2002 assessed burnout in chairs of obstetrics and gynecology; Johns et al. [27] in academic staff of otolaryngology; Cruz [28] in chairs of ophthalmology; and Saleh [22] in orthopaedics. These studies assessed burnout through MBI scale and they revealed from moderate to high levels of emotional exhaustion, depersonalization and lack of personal fulfilment.

Tijdink et al. [26], for the first time, studied burnout and its determinants in an entire nationwide subgroup of medical professors belonging to the university medical centres in The Netherlands through Maslach Burnout Inventory, finding that emotional exhaustion arises in younger medical professors and consequently the risk to develop burnout.

Even if MBI is the most used instrument in researches on burnout, many authors criticized the reliability of this questionnaire and the burnout theory which it is based on. Kristensen et al. [10] Pines et al. [29], and Schutte et al. [30] proposed CBI as a new tool to address the limitations of the MBI.

MBI assesses the phenomenon of burnout according to the original definition by Maslach [2,3] restricted to workers of the human service sector. Later, Maslach and Jackson in 1986 extended the concept of burnout also to inbred aspects of human service work, as the emotional demand [3].

The CBI was introduced by Krinstensen et al. and validated during the Danish longitudinal study PUMA of burnout in employees in the human service sector started in 1997. In this study, the researchers created a new tool to overcome the limitations of MBI and to satisfy the need to measure burnout suitably. Krinstensen et al. criticized the measurement of depersonalization and the reduction of personal accomplishment in the MBI—because they consider these issues as a copying strategy and a consequence, respectively—therefore, these should not be included in the assessment of the burnout phenomenon. In the middle of the 1990s, a new version of MBI was created, the MBI-GS, with new questions added and with more generic items in order to suit to professions even without a close involvement with people [10]. According to Pines [29] and Shirom [31], burnout syndrome is not a result of different dimensions, instead a global phenomenon, characterized by a core of exhaustion, both physical and psychological. This exhaustion develops across different life domains: the personal sphere, the overall work experience, and the specific area of work related to interaction with clients. These domains correspond to the three subscales that compose the CBI, the personal burnout, work-related burnout, and student-related burnout.

The CBI was created to be conform to the evidence of the research on burnout to date [32] and it has been constructed on the central concepts of fatigue and exhaustion, for this reason it seems to fit accurately to the previous definition of burnout by Aronson and Pines from 1988 [33] and to the latest definition of Schaufeli and Greenglass in 2001 [1].

Hence, the CBI consists of a generic part, personal burnout; this section measures the physical and psychological fatigue and exhaustion experienced by the person regardless the working status. The second section is work-related burnout, and it measures the same features, the physical and psychological fatigue and exhaustion experienced by the person but perceived as strictly related to his/her work. This section does not manage to ascribe the fatigue and exhaustion to the working sphere of a person in a scientific way, rather the objective is to define the person’s attribution of these features to the work context [3].

The last scale is about client-related burnout, it assesses the fatigue and exhaustion the person undergoes at work due to their relationship with clients. The generic term ‘clients’ refers to the people with the worker interacts with due to their specific type of work. They can be patients, pupils, students, residents, detainees, etc. [32]. To our knowledge, only one study [34] was conducted to assess burnout in university professors through CBI. This research performed in a Japanese private university and the sample not belong solely to faculty of Medicine.

### Objective

This study aims at evaluating the reliability and validity of the Italian version of Copenaghen Burnout Inventory in a sample of academics belonging to the faculty of Medicine of Sapienza University of Rome. It is the first study in Europe performed on academic professors through administration of this tool. The Italian version of this questionnaire has been validated by Fiorilli et al. in 2015 in an Italian teacher group [5].

## 2. Methods

### 2.1. Study Design

Cross-sectional study, according to the STROBE statement [35] was performed during the period December 2017–February 2018.

### 2.2. Setting

Sapienza University of Rome.

### 2.3. Participants

Table 1 shows information about the population of academics of the Faculty of Medicine and Pharmacy, Sapienza University of Rome teaching in medicine, nursing environmental technician, other health profession degree courses. Participants were invited via e-mail to self-complete an online survey. Ninety-five academics completed the questionnaire (response rate 85%). The mean age of the respondent was 53.6 years, the mean duration of work experience was 16 years, 68.4% of the respondents were women, 45.3% were medicine professors, 28.4% were nursing professors. The academics of the sample were divided in three different groups for the statistical analysis: 33–43 years old, 44–53 years old, and older than 53. These ranges of age seem to correspond better with different stages of typical university career of professors.

### 2.4. Questionnaires

The online questionnaires were composed by Copenhagen Burnout Inventory (CBI) (32), SF12 Health Survey [36], and the Positivity Scale [37]. The request of participation was sent by email and introduced in a neutral way, without any references to “burnout syndrome” to avoid a risk of framing in the answer to the questionnaire. CBI was used for assessing dimension of personal, work-related, and client (student)-related burnout. In detail, the questionnaire was composed in total by 19 items and 3 sections: the personal burnout section consists of six items (e.g., “How often do you feel tired?”, “How often are you physically exhausted?”, “How often do you think: ‘I can’t take it anymore’?”); the work-related burnout was composed of seven items (e.g., “Do you feel worn out at the end of the working day?”, “Are you exhausted in the morning at the thought of another day at work?”, “Does your work frustrate you?”), and the client-related burnout was comprised of another six items (e.g., “Do you find it hard to work with clients?”, “Does it drain your energy to work with clients?”, “Do you feel that you give more than you get back when you work with clients?”). 

The answers that can be given to each item are ‘always or to a very high degree’, ‘often or to a high degree’, ‘sometimes or somewhat’, ‘seldom or to a low degree’ and ‘never/almost never or to a very low degree’, these scores attributed to these answers are 100, 75, 50, 25, and 0% respectively, with an inverse scoring for the fourth item of the work-related burnout, since this refers to the presence of residual energy for family and friends in the spare time. For each scale, it has calculated a total average score.

Health-related quality of life was measured using the Italian version of the Short Form-12 (SF-12) [36]. The SF-12 includes 12 questions from the SF-36 Health Survey. The SF-12 is composed of 12 items: 2 on physical functioning, 2 on physical role, 1 on bodily pain, 1 on general health, 1 on vitality, 1 on social functioning, 2 on emotional role, and 2 on mental health. The items reproduce the physical component summary (PCS) and the mental component summary (MCS) scores. Scores on the SF-36 and SF-12 scales range from 0–100, with higher scores indicating better health. The eight-item positivity scale (PS) [37] was used to measure positivity, defined as the tendency to view life and experiences with a positive outlook. Participants answered eight items on a five-point scale from 1 (‘strongly disagree’) to 5 (‘strongly agree’). The questionnaire addressed sociodemographic variables, such as age, gender, marital status, and number of children, and some basic work variables, such as length of employment, type of work contract, faculty of teaching, commuting.

### 2.5. Statistical Analysis

All analyses, including descriptive statistics and inferential statistics, were carried out using SPSS for Windows 25.0 (IBM, Armonk, NY, USA). A statistically significant difference was accepted at a *p*-value of less than 5%.

Cronbach α coefficients were calculated to assess internal reliability of each domain of the questionnaire.

Descriptive statistics were performed using mean, SD, median, and minimum and maximum for quantitative variables. The frequencies and percentages were computed for qualitative ones. Student’s *t*-test, or Mann–Whitney U test were applied for two groups comparisons, ANOVA and Kruskall–Wallis test for comparisons of more than two groups. The Kolmogorov–Smirnov test was used to verify the normality of the variables. The Pearson’s correlation was computed to estimate the direct or indirect association between the variables. Using the variables from the bivariate analysis, it was developed a multivariate logistic regression model by backward stepwise selection with the three domain of CBI as dependent variables.

### 2.6. Ethical Considerations

The study was approved by the Ethics Committee of the Policlinico Umberto I Rome [project code 4270].

## 3. Results

Cronbach’s α for internal reliability of the three domains were high (0.892 for personal burnout, 0.868 for work burnout, and 0.836 for client burnout) (Table 2).

Univariate analysis (Table 3) show that level of personal burnout (mean score 41.4) in women was significantly higher (*p* = 0.025) than in men. Furthermore, personal burnout level was higher in younger professors, widows, professors with part time contracts (*p* not significant), and in environmental technician professors. Work burnout (mean value 34.3) was definitely higher in younger, part time professors and medicine professors, slightly higher in women (*p* not significant). Student burnout was higher in younger, male, and nursing professors. At the year progresses, work seems to increase only the level of student burnout. Mental scale (MCS), physical scale (PCS), and positivity scale scores were higher among men, environmental technician professors, and contract teachers (Table 4). In multivariate analysis (Table 5) decreasing age (β = −0.263, *p* = 0.001), being a professor in environmental technology (β = −0.120, *p* = 0.098); and low mental (β = −0.263, *p* = 0.020), physical (β = −0.319, *p* ≤ 0.001), and positivity scores (β = −0.237, *p* = 0.031) predict significantly higher personal burnout. Low PCS (β = −0.346, *p* < 0.001); MCS (β = −0.249, *p* = 0.013) and positivity (β = −0.345, *p* = 0.001) scores; fewer years of work (β = −0.269, *p* ≤ 0.001); and being a medical or nursing professor (β = 0.169, *p* = 0.016) predict high work burnout. Low MCS predicts a high level of student burnout.

## 4. Discussion

Results suggest that the Italian version of the CBI is a reliable instrument for analyzing academic populations, as demonstrated by the high values of Cronbach alpha in each scale. In this study, female academics have higher degree of personal burnout. This evidence corresponds with the findings of Taka [34]. It can be speculated that women tend to be prone to experience high degree of personal burnout and the reasons could be related to gender inequality at work or to a weaker role of women in the society. Despite this, most female academics in this study had a favorable contractual position, almost the 75% of the female professors had a permanent contract. In a qualitative review of the burnout literature, Maslach et al. [2] observed that women tend to have a higher degree of emotional exhaustion than men, whereas men tend to be more prone to depersonalization than women.

The women in the sample studied are similar for sociodemographic data to the sample investigated by Taka [34]. The status civil of both sample are comparable in the study of Taka, almost 50% of the women do not have children; in this study, 35% of women did not have sons instead. Civil status and childbearing are similar in the two studies and are important determinants because previous investigations on burnout show that they are protective factors against burnout development [38,39]. In addition to the general work-related aspects, many authors emphasize the influence of sociodemographic characteristics on the development of burnout [2,19,40,41] In CBI questionnaire the first dimension is personal burnout, that investigates personal life of the interviewer beyond the work context and the personality characteristics. Many authors demonstrated that individual attitude to work and character can influence the vulnerability to burnout. CBI is a good tool to assess burnout in people despite their working condition, despite of MBI that measures burnout as a phenomenon merely related to work [10].

It is not possible to make a comparison about the kind of contract and the belonging of particular degree courses because these data have not been showed in the study by Taka.

In the present study, younger and part-time professors are more prone to develop burnout, as demonstrated previously by Tijdink, Cruz, and Gabbe [23,26,28], on the other hand, the increase of years of work seems to only increase the level of student burnout. In the population investigated, younger professors more often have a part-time appointment than older ones, and this pattern reflects the particular nature of university career, as young professors need many years of work to reach a permanent contract. It can be deducted the work precariousness is related to increasing risk to experience burnout. In multivariate analysis conducted in the present study, low mental and physical score of SF12 and low positivity score are related to high degree of burnout in all three domains of CBI. These results are in line with the work of Kristensen [10] where low score in subscales of SF36, the original completed version SF12 derived from, were related to higher level of burnout. The present study shows strong negative correlation between CBI and SF12 and positivity scale; on the other side, Fiorilli [5] analyzed the correlation between CBI and work-engagement and self-efficacy measures, founding the same negative correlation.

Academics belonging to medicine and nursing degree courses have significantly higher rate of work burnout. It could be speculated that these workers can be more disposed to experience burnout for contemporary or previous load of work in patient care sector [22,23].

Every subscale of the CBI correlates with the two other subscales composing the questionnaire, as already demonstrated by Kristensen in the PUMA study and by Fiorilli in the study of validation of the Italian version of CBI by Fiorilli. These findings assess the convergent validity of the CBI. Academics have a central role in the society, their wellbeing, which can be impaired when they experience burnout, is a remarkable issue, since they teach future professionals and they train them. Not only the degree of teaching, but also the quality of research in the universities is assured by their work. For this reason, the professors and people that manage and supervise the university system should be made aware of this phenomenon. Different strategies of support for academics should be actuated in order to control the phenomenon, it seems obvious that the approach to counteract burnout cannot be only one. It has been demonstrated that consultation service is useful in workplaces where burnout rate is high [34,42,43]. The implementation of workshops to teach academics to recognize and to control the risk of burnout at work and in their private lives could also be helpful.

### Study Limitations

A first limitation can be represented by the relatively small amount of the sample. This study has been conducted among Academics belonging to only one University, and it aimed at validating CBI in professors of Medicine Faculty. For this reason, the number of the sample is not so big. Moreover, the cross-sectional nature of study doesn’t allow to make inference on causality or temporal order of variables.

Furthermore, it cannot be excluded a response bias, even if both workers that experience higher degree of burnout and those that are less affected, are supposed to be reluctant to participate for opposite reasons, as a sense of waste of time to use to other tasks of work. Moreover, as this study was conducted by a group of research of the same university, it is possible to expect a response bias due to some reluctance to answer sincerely to this kind of survey by the participants, despite of the preservation of confidentiality.

For respondents that work also in patient care this aspect can influence and interfere with the score obtained in the three different subscales of the CBI.

Regarding the strengths of the study to our knowledge, at the time this paper is written, this is the first study to assess burnout in academics of Medicine Faculty by CBI and the first time the CBI has been used and validated in this category of workers in Italy. According to the latest researches on burnout and its theoretical model, even if it has been estimated that the MBI has been used in 90% of the scientific studies [43], CBI could be considered more reliable tool to evaluate this phenomenon [5,6]. Moreover, in this study the population of academics belonging to the degree courses considered to was recruited with high response rate: the 85% of academics fulfilled the online questionnaire, so so the representativeness of the sample was considered preserved.

This study reveals a high alpha Cronbach coefficient, it proves a high internal reliability as previously found by Kristensen. The results show the same alpha Cronbach coefficient in Personal burnout as it Fiorilli et al [5] found in their investigation, and even a higher coefficient in Work-related burnout and Student-related burnout.

The survey was performed by online questionnaires that can be considered as reliable as live questionnaires. Moreover, this kind of tools allows the respondents to fulfill the questionnaire at home, in a more comfortable setting and when the interviewer feels at ease, without any kind of pressure related to his/her work context.

The study was conducted in December- February to avoid an underestimation or overestimation of the phenomenon due to the peculiar organization of the academic year, with a long period of stop for summer holidays that in our country last from the end of July to the beginning of September. 

This study reveals a good reliability of the CBI in academics, further researches through a multicentre study should be carried out to assess the burnout syndrome in this category of workers.

## 5. Conclusions

This study reveals that burnout is present among academics, this should be considered an opportunity to further assess the phenomenon by looking closer at stressors and risk factors that can develop it. University organizations, in particular public universities, should try to develop strategies to avoid the rise of this syndrome. Results suggest that the Italian version of the CBI, never used in Europe in this kind of workers, is a reliable toll. Further research should focus on the prevalence of burnout among academics for the effect on their wellbeing and consequences on teaching, publishing, and University performance.

## Figures and Tables

**Table 1:** Characteristic of the whole population.

Variables		N (%) or Mean (SD)
Gender	Male	49 (51.6)
	Female	46 (48.4)
Age	33–43	16 (16.8)
	44–53	26 (27.4)
	>53	53 (55.8)
Civil status	Single	15 (15.8)
	Married/cohabitant	70 (73.7)
	Widow/widower	2 (2.1)
	Divorced	8 (8.4)
Teaching	Medicine	43 (45.3)
	Nursing	27 (28.4)
	Environmental technicians	17 (17.9)
	Other health professions	8 (8.4)
Type of work	Part time professor	13 (13.7)
	Contract teacher	21 (22.1)
	Full time Academics	61 (64.2)
Time to go to work	<15 min	20 (21.1)
	16–30 min	39 (41.1)
	>30 min	36 (37.9)
Sons	0	28 (29.5)
	1	23 (24.2)
	2	38 (40.0)
	3	6 (6.3)
Year of work	1–10	31 (32.6)
	11–20	44 (46.3)
	>20	20 (21.1)

**Table 2:** Results of the Cronbach’s Alpha for the questionnaire dimensions.

Dimension	Alpha
Personal burnout	0.892
Work burnout	0.868
Client burnout	0.836
Personal, work, and client burnout	0.918

**Table 3:** Univariate analysis for the three dimensions of the Copenhagen Burnout Inventory.

Variables	Personal Burnout		Work Burnout		Client (Student) Burnout	
	Mean	*p*	Mean	*p*	Mean	*p*
Gender						
Male	41.86	0.025	47.38	0.820	49.22	0.653
Female	54.54		48.66		46.70	
Civil status						
Single	54.73	0.665	53.33	0.842	45.77	0.964
Married/cohabitant	46.92		46.54		48.61	
Widow/widower	57.50		50.50		40.75	
Divorced	42.44		50.19		48.63	
Teaching						
Medicine	48.49	0.945	50.55	0.504	47.52	0.587
Nursing	47.76		50.22		52.43	
Environmental technicians	49.65		43.21		47.18	
Other health professions	42.69		37.00		37.38	
Type of work						
Part time professor	56.46	0.060	54.77	0.477	55.23	0.381
Contract teacher	36.07		43.02		42.00	
Full time Academics	50.30		48.27		48.52	
Time to go work						
<15 min	41.23	0.403	50.08	0.928	53.00	0.216
16–30 min	51.41		47.65		42.13	
30–60 min	48.07		47.22		51.58	

**Table 4:** Correlation matrix.

Variables	1	2	3	4	5	6	7	8	9
Age (1)	1								
Year of work (2)	0.587 **	1							
PCS12 (3)	−0.125	−0.218 *	1						
MCS12 (4)	0.102	0.080	0.400 **	1					
Pos Scale (5)	−0.05	−0.24 *	0.38 **	0.70 **	1				
Children (6)	0.28 **	0.00	0.04	0.12	0.09	1			
Personal burnout (7)	−0.27 *	−0.00	−0.42 **	−0.58 **	−0.51 **	−0.19	1		
Work burnout (8)	−0.24 *	−0.07	−0.44 **	−0.59 **	−0.60 **	−0.09	0.78 **	1	
Client burnout (9)	−0.07	−0.04	−0.26 *	−0.28 **	−0.23 *	0.01	0.40 **	0.53 **	1

** *p* < 0.01; * *p* < 0.05.

**Table 5:** Multivariate analyses for the three dimensions of the Copenhagen Burnout Inventory.

Variables	Personal Burnout	Work Burnout	Client Burnout
	Beta (*p*)	Beta (*p*)	Beta (*p*)
Gender F ^	0.14 (0.047)		
Age	−0.26 (0.001)		
Teaching * Medicine Nursing	0.12 (0.098)	0.17(0.016)	
Civil status ° Married		−0.17 (0.017)	
Year of work		−0.27 (<0.001)	
MCS	−0.26 (0.020)	−0.25 (0.013)	−0.39 (<0.001)
PCS	−0.32 (<0.001)	−0.35 (<0.001)	
Positivity scale	−0.24 (0.031)	−0.35 (0.001)	
R^2^	0.55	0.62	0.14

^ reference = male; * ref = other health profession; ° ref = single.
